# Role of 3D electro-anatomical mapping on procedural characteristics and outcomes in pulsed-field ablation for atrial fibrillation

**DOI:** 10.1093/europace/euae075

**Published:** 2024-03-25

**Authors:** Patrick Badertscher, Teodor Serban, Corinne Isenegger, Philipp Krisai, Gian Voellmin, Stefan Osswald, Sven Knecht, Christian Sticherling, Michael Kühne

**Affiliations:** Department of Cardiology, University Hospital Basel, Petersgraben 4, 4031 Basel, Switzerland; Cardiovascular Research Institute Basel, University Hospital Basel, Petersgraben 4, 4031 Basel, Switzerland; Department of Cardiology, University Hospital Basel, Petersgraben 4, 4031 Basel, Switzerland; Cardiovascular Research Institute Basel, University Hospital Basel, Petersgraben 4, 4031 Basel, Switzerland; Department of Cardiology, University Hospital Basel, Petersgraben 4, 4031 Basel, Switzerland; Cardiovascular Research Institute Basel, University Hospital Basel, Petersgraben 4, 4031 Basel, Switzerland; Department of Cardiology, University Hospital Basel, Petersgraben 4, 4031 Basel, Switzerland; Cardiovascular Research Institute Basel, University Hospital Basel, Petersgraben 4, 4031 Basel, Switzerland; Department of Cardiology, University Hospital Basel, Petersgraben 4, 4031 Basel, Switzerland; Cardiovascular Research Institute Basel, University Hospital Basel, Petersgraben 4, 4031 Basel, Switzerland; Department of Cardiology, University Hospital Basel, Petersgraben 4, 4031 Basel, Switzerland; Cardiovascular Research Institute Basel, University Hospital Basel, Petersgraben 4, 4031 Basel, Switzerland; Department of Cardiology, University Hospital Basel, Petersgraben 4, 4031 Basel, Switzerland; Cardiovascular Research Institute Basel, University Hospital Basel, Petersgraben 4, 4031 Basel, Switzerland; Department of Cardiology, University Hospital Basel, Petersgraben 4, 4031 Basel, Switzerland; Cardiovascular Research Institute Basel, University Hospital Basel, Petersgraben 4, 4031 Basel, Switzerland; Department of Cardiology, University Hospital Basel, Petersgraben 4, 4031 Basel, Switzerland; Cardiovascular Research Institute Basel, University Hospital Basel, Petersgraben 4, 4031 Basel, Switzerland

**Keywords:** Pulsed-field ablation, Mapping, Atrial fibrillation, Pulmonary vein isolation

## Abstract

**Graphical Abstract:**
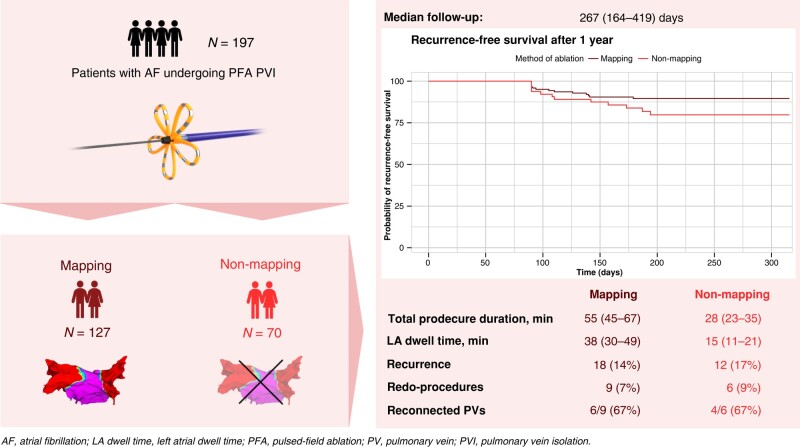

Pulsed-field ablation (PFA) holds the promise of ushering a new era in the treatment of atrial fibrillation (AF). Initial studies have reported positive procedural outcomes and potentially more durable ablation lesions compared to traditional ablation techniques such as radiofrequency and cryoballoon pulmonary vein isolation (PVI).^1^ While the utilization of dedicated 3D electro-anatomical mapping (EAM) systems for catheter ablation is crucial using radiofrequency, its potential benefits in PFA remain uncertain.^2^

Thus, our objective was to compare the procedural characteristics and outcomes of patients undergoing PFA for PVI with mapping and with no mapping.

We prospectively enrolled patients undergoing their first PVI at a tertiary referral centre. In brief, the 31 mm FARAWAVE PFA catheter (Boston Scientific, Marlborough, MA) was inserted into the left atrium via a single transseptal puncture. The PFA catheter was visualized during the procedure within the EAM system. Procedural details have been previously described.^3^ In patients undergoing PVI with mapping, a multipolar mapping catheter (OCTARAY, Biosense Webster) was used to create voltage maps during sinus rhythm pre- and post-ablation. In the non-mapping group, PVI was confirmed by assessing the entrance block via the PFA catheter. The first 30 cases with mapping were excluded to account for a potential learning curve. The study was carried out according to the principles of the Declaration of Helsinki and was approved by the local ethics committees.

A total of 197 consecutive patients were included {age 65 [interquartile range (IQR) 58–72] years; left ventricular ejection fraction (LVEF) 58 [IQR 52–63] %; indexed left atrial volume 40 [IQR 35–44] mL/m^2^}. Among these, 127 patients (64%) underwent PVI with mapping and 70 patients (36%) with no mapping. Baseline characteristics were similar between the groups. The median procedure duration, left atrial dwell time, and the fluoroscopic time for the mapping vs. the non-mapping group were 55 [IQR 45–67] min vs. 28 [IQR 23–35] min (*P* < 0.001); 38 [IQR 30–49] min vs. 15 [IQR 11–21] min (*P* < 0.001); and 11 [IQR 9–14] min vs. 8 [IQR 7–11] min (*P* < 0.001), respectively. In the mapping group of 9% (11/127 patients), at least 1 PV was incompletely isolated and required additional applications. Acute PVI success was 100%. There were two complications in the mapping group (one stroke, one coronary artery air embolism), and none were observed in the non-mapping group. The recurrence rate of atrial arrhythmias during a median follow-up of 267 [IQR 164–419] days was 14% in the mapping group and 17% in the non-mapping group (*P* = 0.728).

Our findings corroborate previous studies assessing the impact of mapping on patients undergoing cryoballoon ablation.^4,5^ Consistent with previous findings, we did not find any objective added benefit by using mapping for PFA–PVI. Given the additional cost of mapping, the routine use of a 3D EAM system in all first-time PVI–PFA cases may not be justified. While the use of mapping, like intracardiac echocardiography, might be reasonable to support the learning curve for vein identification, assessment of catheter–tissue contact, or post-ablation testing, its benefit for routine long-term use must be balanced against a potential safety bonus for a PFA-only approach by significantly shorter LA dwell times and elimination of catheter exchanges via the transseptal sheath. We previously demonstrated the use of a systematic pacing protocol for endpoint verification via the PFA catheter.^1^ The impact of the identification of incompletely isolated PV can only be fully assessed in a randomized controlled trial using re-mapping protocols.

Our study has important limitations, including the non-randomized study design and the small sample size. Our findings cannot be extrapolated for PFA systems with integrated 3D EAM capabilities.

In conclusion, the use of a pentaspline PFA system with no mapping was associated with a significant decrease in procedural characteristics, while AF recurrence was not significantly different. The routine use of mapping for PFA–PVI may not be needed.

## Data Availability

All relevant data are within the manuscript and its supporting information files.
